# Molecular Phylodynamics of the Heterosexual HIV Epidemic in the United Kingdom

**DOI:** 10.1371/journal.ppat.1000590

**Published:** 2009-09-25

**Authors:** Gareth J. Hughes, Esther Fearnhill, David Dunn, Samantha J. Lycett, Andrew Rambaut, Andrew J. Leigh Brown, on behalf of the UK HIV Drug Resistance Collaboration

**Affiliations:** 1 Institute of Evolutionary Biology, School of Biological Sciences, University of Edinburgh, Edinburgh, Scotland, United Kingdom; 2 Medical Research Council Clinical Trials Unit, London, United Kingdom; Fred Hutchinson Cancer Research Center, United States of America

## Abstract

The heterosexual risk group has become the largest HIV infected group in the United Kingdom during the last 10 years, but little is known of the network structure and dynamics of viral transmission in this group. The overwhelming majority of UK heterosexual infections are of non-B HIV subtypes, indicating viruses originating among immigrants from sub-Saharan Africa. The high rate of HIV evolution, combined with the availability of a very high density sample of viral sequences from routine clinical care has allowed the phylodynamics of the epidemic to be investigated for the first time. Sequences of the viral protease and partial reverse transcriptase coding regions from 11,071 patients infected with HIV of non-B subtypes were studied. Of these, 2774 were closely linked to at least one other sequence by nucleotide distance. Including the closest sequences from the global HIV database identified 296 individuals that were in UK-based groups of 3 or more individuals. There were a total of 8 UK-based clusters of 10 or more, comprising 143/2774 (5%) individuals, much lower than the figure of 25% obtained earlier for men who have sex with men (MSM). Sample dates were incorporated into relaxed clock phylogenetic analyses to estimate the dates of internal nodes. From the resulting time-resolved phylogenies, the internode lengths, used as estimates of maximum transmission intervals, had a median of 27 months overall, over twice as long as obtained for MSM (14 months), with only 2% of transmissions occurring in the first 6 months after infection. This phylodynamic analysis of non-B subtype HIV sequences representing over 40% of the estimated UK HIV-infected heterosexual population has revealed heterosexual HIV transmission in the UK is clustered, but on average in smaller groups and is transmitted with slower dynamics than among MSM. More effective intervention to restrict the epidemic may therefore be feasible, given effective diagnosis programmes.

## Author Summary

Since 1995, HIV among heterosexuals in the UK increased to the point where the total number of heterosexuals infected with HIV, predominantly of non-B subtypes, exceeds the number of HIV-positive homosexual men. To understand the dynamics of this epidemic, we have applied the novel technique of phylodynamics to the analysis of viral sequences taken in the course of routine clinical care from approximately 40% of the HIV-infected heterosexual population in the UK. Phylodynamics reconstructs the pattern of viral sequence divergence in time, revealing the size of transmission clusters and the dynamics of transmission within them. Of 11,071 patients studied, 296 were linked to at least two others in the UK. There were 8 clusters comprising 10 or more individuals among these, yielding a total of 143 or 5% of all individuals with links, much lower than seen earlier among homosexual men (25%). Viral transmissions within clusters also occurred less rapidly, only 2% being dated to the first 6 months of infection, compared to 25% among homosexual men. Overall, transmission clusters exist in the UK heterosexual HIV epidemic but they are generally smaller than among homosexuals; onward transmission occurs less rapidly and is not associated with acute HIV infection.

## Introduction

HIV infection was first detected in the United Kingdom (as AIDS) in 1981–2 [1] among MSM. Early outbreaks with UK sources include Scottish IDUs dated to 1983 [2] and haemophiliacs to 1984 [3]. All strains isolated initially were of the B subtype, both in MSM and IDUs [4] and also in the small number of individuals infected through heterosexual contact during that decade [5]. However within 10 years, multiple subtypes had been detected within the UK [6].

From the mid 1990s increasing numbers of HIV infections in the UK were being found in heterosexuals, until the current situation was attained whereby this risk group comprises the majority of new HIV diagnoses [7]. This increase coincided with increasing immigration from southern and Eastern Africa, particularly from South Africa, Uganda and Zimbabwe [8]. Genetic characterisation of viruses from infected heterosexuals revealed that while subtype B was still observed in the majority of samples obtained during 1996/7 [9], by the year 2000, subtype C was most common (35%) with subtype A at 15%, reflecting the main subtypes in those countries. Subtype B was present in only 25% of individuals [10]. Thus, the heterosexual risk group in the UK has become strongly associated with non-B HIV subtypes. Recently there has been some evidence of limited crossover among risk groups with a study of over 5000 patients from London reporting 2 small clusters of subtype A (n = 21) among MSM, of whom approximately 50% of individuals were white [11].

We have applied recently developed methods of molecular phylodynamics to the analysis of partial HIV pol gene sequences obtained during routine clinical care from over 2000 MSM attending a single large clinic in London [12]. We showed that 25% of individuals whose virus showed a link to at least one other individual in the study were in fact linked to 10 or more others. Using relaxed clock approaches [13] we found that 25% of transmissions within these clusters took place within a maximum of 6 months after infection. This suggested that the elevated risks of transmission associated with acute HIV infection could be important for driving a significant component of the HIV epidemic among MSM.

In this study we have analysed the entire dataset of individuals infected with non-B subtypes of HIV and receiving clinical care within the UK who are represented in the UK HIV Drug Resistance Database. The overwhelming majority (95%) of non-B subtype HIV in this dataset is associated with heterosexual transmission and 83% with Black-African ethnicity [14]. Since 2003 in the UK, a baseline HIV genotyping assay has been recommended when antiretroviral therapy is initiated and accordingly a large proportion of sequences within the database have been obtained prior to therapy. Non-B subtype HIV pol sequences were available from over 11,000 individuals for this study: for comparison the estimated number of HIV-infected Black African and Caribbean individuals in the UK was 24,000 in 2007 [7]. We therefore estimate we have analysed almost 40% of the UK heterosexual HIV-infected population.

## Results

### Detection of transmission clusters

From the sequence dataset representing over 25,000 subjects, non-B subtypes were identified mainly using the REGA method [15], with additional information from ad hoc phylogenetic analysis (see Methods). Due to the limited number of subtypes other than A and C, these other non-B subtypes were grouped for analysis. This gave datasets of the following sizes: for subtype A, *N* = 1581; for C, *N* = 6096 and for other non-B subtypes, *N* = 3394. Within these groups, the initial subset of sequences linked to at least one other was selected from all pairwise comparisons using the threshold of 4.5% nucleotide similarity at third codon positions [12]. This identified sequences from 367 patients infected with subtype A, 1372 infected with subtype C and 1035 infected with other non-B subtypes, a total of 2774 individuals.

The datasets were then modified by removal of codons associated with drug resistance (see Methods) and Bayesian MCMC phylogenetic analysis was performed on subtype A and subtype C separately. In the resulting trees, 4 subtype A and 14 subtype C phylogenetic clades of ≥10 individuals were identified with a posterior probability of 1 (Figures S1 & 2). This corresponds to 25% of the subtype A closely-related sequences and 21% of the subtype C closely-related sequences. A similar analysis was performed on the 1035 sequences from other non-B subtypes. In the last case, the main fully supported clades reflected subtype divisions and were unrelated to transmission patterns. However, from within the main subtype splits we were able to identify 7 fully supported subtrees of ≥10 individuals for further analysis (Figure S3).

**Figure 1:**
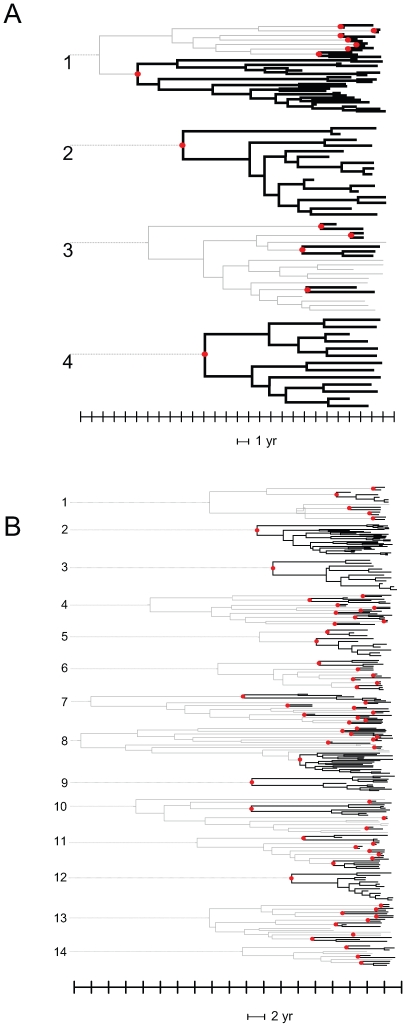
Time-scaled Bayesian MCMC phylogenies of clusters of ≥10 patients. Red dots indicate the most recent common ancestor (MRCA) of UK transmission clusters as defined against analysis with global diversity. The scale bar is in calendar years. Grey lines indicate non-UK-based segments of the phylogeny, black lines indicate UK-based lineages. A) Subtype A. Scale bar indicates calendar years. B) Subtype C. Scale bar indicates 2 calendar years.

**Figure 2:**
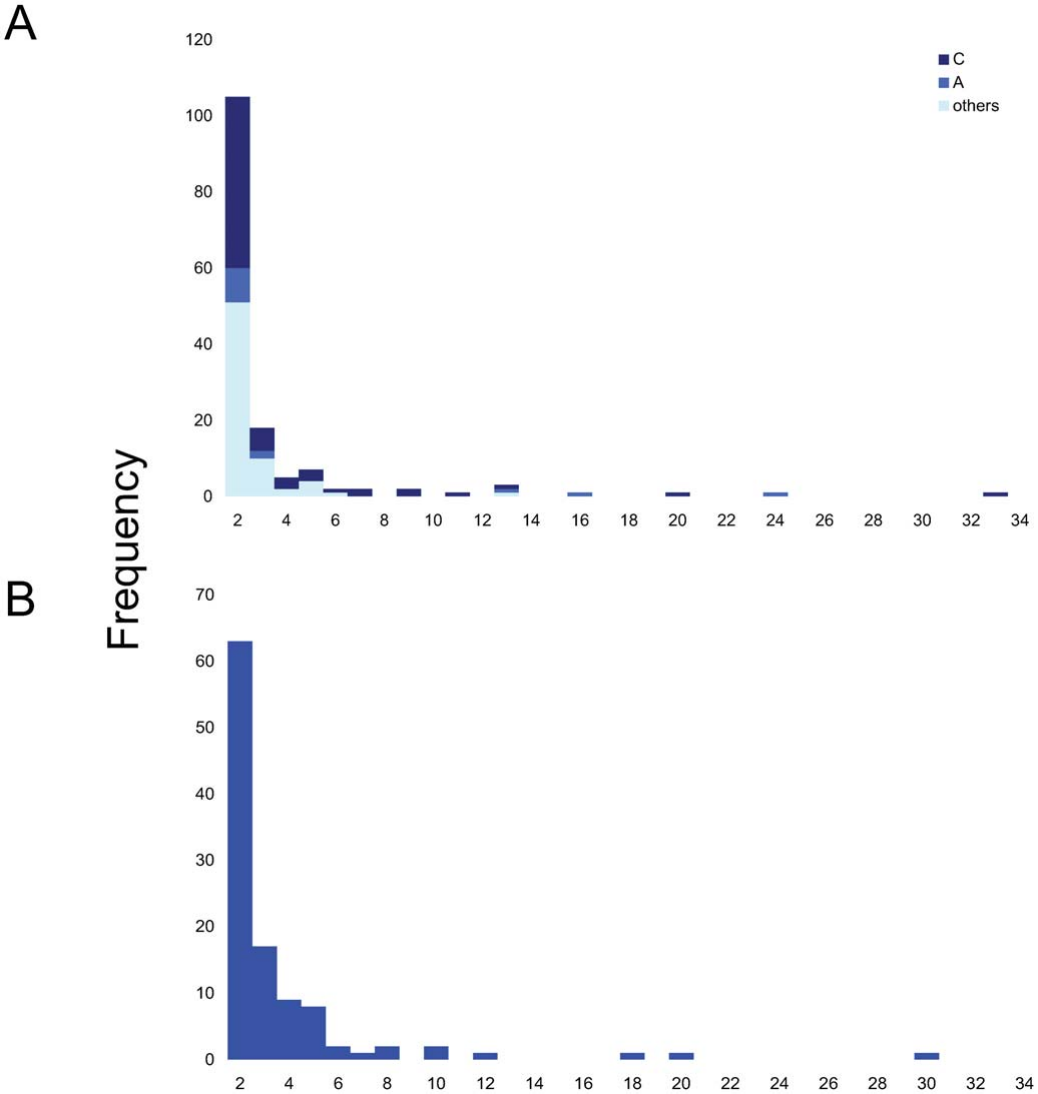
Distribution of cluster size. Frequency of UK-based clusters, as defined in the text, of size 2 or higher, identified by subtype. A) Non-B subtypes (this study). B) Subtype B [12].

Unlike the case for the subtype B sequences previously studied [12], the clustering of non-subtype B sequences includes patient linkage outside of the UK. We therefore performed further analyses in which the nearest sequences to each cluster from the global HIV database were included. This leads to the breakdown of a number of clades through the inclusion of sequences from outside the UK within what were previously monophyletic groups (Figure 1A, 1B & S4). The resulting distribution of cluster size is shown in Figure 2. Including the closest sequences from the global HIV database left 296 individuals that were in UK-based groups of 3 or more individuals. Large clusters still comprise a significant proportion of patients with a link to at least one other. The largest for subtype A was a cluster with 24 individuals and that for subtype C was one of 33 individuals. The percentage of sequences found in clusters ≥10 individuals was 14% (subtype A); 6% (subtype C) and 1% (others), respectively. A total of 143 of the original 2774 (5%) individuals were found in large clusters, although these comprised 48% of individuals within UK-based groups of 3 or more.

**Figure 3:**
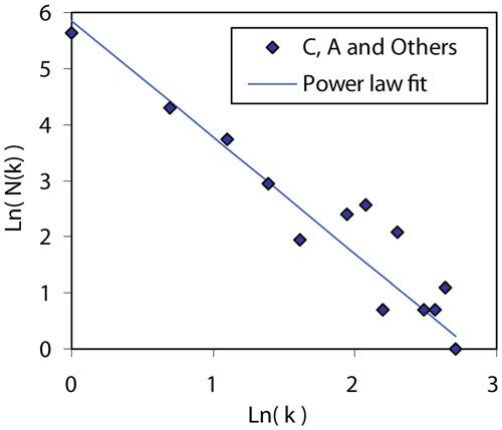
Power law plot of UK-based non-B subtype clusters. Log-log plot of numbers of individuals with k contacts (N(k)) against the number of contacts (k). Individuals are assumed to be in contact within the clusters only if the time to the most recent common ancestor of their virus sequences is less than or equal to 5 years. The best fit to a power law (straight line in log-log space) has R^2^ = 0.95 (95% CI: 0.84–0.99), p<10^−6^, and shape parameter (negative gradient) α = 2.1.

In this and our previous study of subtype B sequences, the distribution of individuals in clusters strongly suggested a power law relationship indicative of a scale-free network. With the additional data available we have examined the fit of a power law to the non-B subtype data. The goodness of fit to a power law varies with the maximum time depth allowed for clusters. We have used the date of sampling to limit the time depth and having considered a range of values (Figure S5), find that restricting the analysis to subclusters with a maximum depth of 5 years reveals a very good fit (Figure 3; R^2^ = 0.95; p<10^−6^; α = 2.1).

### Transmission intervals

We previously made use of the statistically rigorous approach of relaxed-clock phylogenetics implemented in BEAST to obtain estimates, and highest posterior density distributions, of dated nodes within clusters [12]. Each sequence is obtained from a different patient so from the internode interval we can infer maximum, estimates of inter-transmission intervals. Any missing data in the form of additional individuals in the network would lead to shorter average transmission intervals.

**Figure 4:**
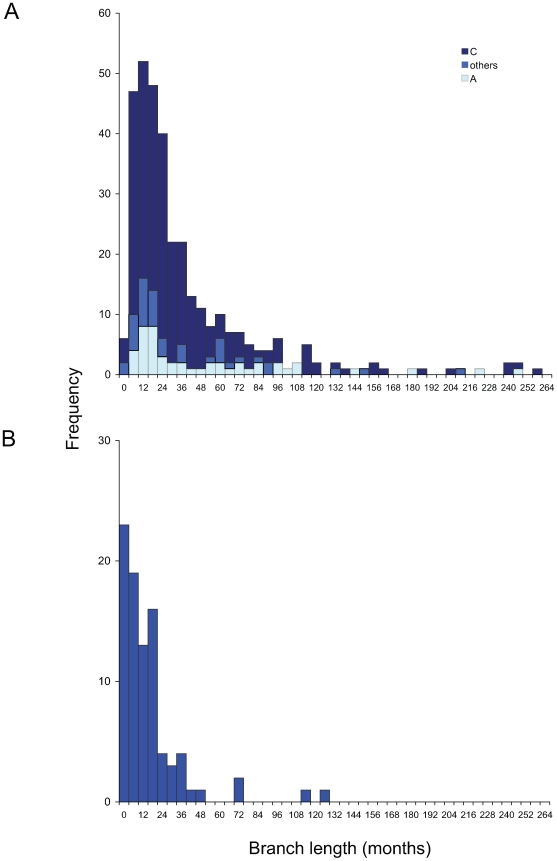
Histogram of internal branch lengths from time-scaled trees representing minimum transmission intervals. A) Non-B subtype UK transmission clusters, as defined in the text. B) Subtype B transmission clusters from MSM in London [12].

For all time-scaled subtrees of all UK-based groups containing ≥3 individuals (296 individuals in total), determined as described above, the internode distances were estimated (Figures S6, S7, S8). These yielded maximum estimates of transmission intervals for UK-based non-B clusters whose medians were 32 months (subtype A) and 25 months (C), respectively and 22 months for other subtypes (Figure 4A). Overall, for non-B HIV the median transmission interval for UK-based groups was 27 months.

The proportion of transmission intervals in the first 6 months of infection was 0%, 2% and 5% for subtypes A, C and others, respectively, giving 2% overall. The proportion of transmission intervals between 6–36 months after infection for the non-subtype B clusters were: 53% (subtype A), 68% (subtype C) and 56% (others) with an overall proportion of 62%. In this population therefore, the possible heightened risk of transmission associated with acute infection appears not to play a significant role in the epidemic (Figure 4).

## Discussion

We have retrospectively investigated the dynamics of the developing heterosexual HIV epidemic in the UK by applying Bayesian phylogenetic analysis to anonymised viral sequences obtained in the course of routine clinical treatment. The high level of representation in the UK HIV Drug Resistance Database (over 40% of the estimate of the relevant risk group) has permitted a detailed analysis of the level of clustering, the distribution of cluster size and the distribution of the interval between transmissions for non-B subtype sequences. After screening out non-UK associations, we have found that among probable UK-based infections, 14% of subtype A sequences were found in clusters ≥10 individuals, with 6% of subtype C and 1% for others, although these percentages increase sharply (to a total of 48%) if the denominator is restricted to the 293 individuals within UK-based clusters of 3 or more. That this would suggest that individuals within a UK-based cluster of any size are very likely to be in a large one is a striking conclusion as all likely confounding factors (such as immigration of concordant families) might increase the numbers of pairs, and perhaps clusters of 3 individuals but not of clusters of 10 or more, and therefore would decrease the proportion in large clusters. Despite the different geographical origin of HIV-1 subtypes, large clusters were observed in both subtype C (33 members) whose primary origin would be southern Africa, and subtype A (24 members) which is primarily associated with East Africa, suggesting no major distinction in the structure of the epidemic among communities from different countries.

We explored the epidemic in these groups in greater detail by using time-resolved phylogenies to analyse the dynamics of transmission within clusters, adopting a relaxed molecular clock [13]. As each sequence is obtained from a different infected individual we take the internode interval as a maximum estimate of the time between transmissions [12]: missing data, in the form of individuals within the transmission network who were not sampled, would always reduce this estimate. Taking this approach a median estimate of the time between transmissions of 27 months was observed overall for non-B subtypes (32, 25 and 22 months for subtypes A, C and other, respectively). This approach also allowed the estimation of the proportion of transmissions within defined intervals after infection: overall just 2% of transmissions in this population were estimated to occurr within 6 months or less (0%, 2% and 5% for A, C and other subtypes, respectively).

In an earlier study of the phylodynamics of HIV in an MSM population attending a large clinic in London we observed a much higher frequency of linkage between individuals with 25% of those with a connection to at least one other being found in large clusters [12]. Among these MSM the median transmission interval within clusters, estimated in the same way, was almost half that for the heterosexual population studied here, at 14 months, and 25% of transmissions within clusters occurred within 6 months of infection. Nevertheless, the shape of the distribution of cluster size was similar between the two groups. The overall proportion of transmission intervals between 6–36 months after infection for the heterosexual clusters, 62%, is very similar to that estimated for the MSM dataset (63%; Figure 2B). While there is an extended right-hand tail of the transmission interval distribution for non-subtype B UK transmission clusters (Figure 2A) this is likely to be due in part to the inclusion of a residue of non-UK based distantly linked sequences which were not identified by the global diversity screen. In a recent study of patients selected in primary transmission in Quebec, Brenner et al. [16] indicated that while 28% of MSM diagnosed early in infection were part of transmission clusters involving 5 or more individuals, only 13% of non-B subtype infections (mostly heterosexual) were in clusters.

The observed differences between MSM and heterosexuals in inter-transmission intervals could reflect real differences in the dynamics of the epidemics in different risk groups. In this study a possible cause of such a distinction could have been a systematic difference between them, for example in the sampling of the population if there were many more missing individuals from the heterosexual clusters. At the most basic level this would appear to work in the opposite way, as the earlier MSM study was restricted to individuals attending a single clinic in London [12], while the analysis presented here derived from population surveillance of all HIV-infected individuals receiving treatment in the United Kingdom. As indicated earlier (see Introduction), these results reflect approximately 40% of the HIV-infected Black African population. In contrast, the earlier study analysed 2126 individuals sampled from approximately 11,000 MSM receiving care in London (www.hpa.org.uk), i.e. ∼20% of those receiving care and perhaps 10–15% of all MSM in London. We therefore have approximately 3–4 fold greater coverage of the of African-derived HIV in the UK in this study than of MSM in London previously.

Another possible source of bias could lie in the frequency of testing. The possibility of higher awareness and/or access to HIV-related care among MSM than among the predominantly immigrant HIV-infected heterosexual group could in principle have led to a shorter time between infection and diagnosis. If this also led to a shorter time between infection and initiation of antiretroviral therapy then the period of opportunity for transmission could be reduced. Time of infection is unknown for most of the patients studied so we investigated this possibility by using CD4 counts at the time of diagnosis as a proxy for the average time since infection (Text S1, Table S1). In agreement with Stöhr et al. [17], we conclude that there is little difference between the heterosexual and MSM groups in the UK (Figure S9): the 10% difference we observe in CD4 count at treatment between subtypes C and B cannot explain the observed 50% difference in the median inter-transmission interval.

Following the observations of Liljeros et al. [18] that human sexual networks based on contacts within the last year have the properties of scale free networks, we have examined the distribution of the size of transmission clusters among heterosexuals in the UK and find an excellent fit to a power law, consistent with a scale-free network (Figure 3). Inference from viral sequence data is not direct and as discussed in detail earlier [12], it is important to recognise that the viral transmission network and the sexual network are not the same in a chronic infection such as HIV: a series of transmissions could derive from a single individual rather than as onward transmissions from their sexual contacts. The transmission network is a subgraph of the sexual network but clearly both incorporate a time dimension; the network that fits a power law was that described in terms of sexual contacts in the last year [18] and is smaller than the lifetime network. Here we tested several time depths and found that the best fit was obtained with a limit of 5 years, and the value of the shape parameter α, was estimated at 2.1 (Figure 3 and S5), close to estimates obtained by Liljeros et al. [18] The greater time depth reflects the substantial delay that is usual between infection, diagnosis and the onset of antiretroviral therapy, which would have been the indication for a HIV genotype test from which our sequences are derived. While nodes in a sexual network and nodes in a transmission network cannot be directly equated, the distribution in time of the latter is clearly bounded by the former. On the other hand, the relationship of the sexual network to the transmission network is determined by the probability of transmission per contact which varies greatly and is difficult to estimate [19]. Therefore a quantitative description of the transmission network for a population can provide critical information for modelling the epidemiology of HIV transmission.

The degree of clustering deduced from heterosexual population differs from that found previously for MSM and there is a substantial difference in the dynamics. While it is generally recognised that concurrent partnerships form the greatest potentiating factor for HIV and other STIs, the difference between these risk groups suggests either a longer interval between partner change, or a lower per-contact risk of transmission in heterosexuals. With very few inter-transmission intervals below 6 months it is unlikely that the elevated viral load associated with acute infection [20] plays a significant role in the UK heterosexual epidemic. The slower dynamics of the heterosexual epidemic thus offer more opportunity for successful intervention, but it is essential that diagnosis is achieved as early as possible.

## Methods

### Patients

The patient data derived from the 25631 patients in the UK HIV Drug Resistance Database (www.hivrdb.org) as at 2007, who had been recruited over the previous 10 years (Text S1, Figure S10). Of the patients reported on here with non-B subtype HIV 5777 (76%) were recruited from London, 797 (11%) from Manchester, 549 (7%) from the rest of northern England and Scotland and 463 (6%) from Birmingham and the Midlands.

Ethical approval for this work was given by the London Multicentre Research Ethics Committee (MREC/01/2/10; 5 April 2001).

### Sequences

Where multiple sequences were present for patients within the database the oldest sequence was selected. Sequences were aligned using the sequence alignment tool in HyPhy [21] with problematic sequences aligned manually by eye. The final alignment was 1554 nucleotides (nt) in length (concatenated full-length protease [PR] and partial reverse transcriptase [RT] coding sequences) with individual sequences ranging from 791–1536 nucleotides (median 1269), according to genotyping method. HIV-1 subtype was determined using REGA [15] and HIVdb (http://hivdb.stanford.edu). Sequences for which the two methods yielded discordant results were additionally assessed phylogenetically (using NJ trees created in PAUP* under the HKY85 nucleotide substitution model [22]) for clustering with sequences representing the 10 major subtypes within the dataset (A, B, C, CRF02_AG, D, G CRF06_cpx, F, H, J). Unique inter-subtype recombinants which might confound the phylogenetic analysis were eliminated at this stage. Within-subtype recombination, which would remain undetected, would introduce artifactually long branches and could have the effect of removing some individuals from clusters that they belonged to. Only limited numbers of subtypes other than A and C were found; these other non-B subtypes were grouped together for analysis. Clinical and epidemiological data was available for those patients recruited to the United Kingdom Collaborative HIV Cohort (UK CHIC) [23]; A: 620 (39%), C: 1387 (23%), other non-B: 1207 (36%).

### Identification of transmission clusters

Phylogenetic analysis was performed as described earlier [12], initially removing 39 codons associated with antiretroviral resistance creating an alignment of 1437 nucleotides in length. Identification of clades likely to contain UK transmission clusters was performed using Bayesian Monte Carlo Markov Chain (MCMC) approach [24] with the HKY85 model of nucleotide substitution with gamma distribution of rate variation (Γ). The HKY model was used because the very large size of the trees being generated meant the more complex GTR model would lead to an excessive computation time. In this study the greatest interest lies in the most closely related sequences where the difference in performance between these two models is least. Trees were rooted using a subtype G outgroup taken from the UK HIV Drug Resistance Database. Due to the size of the alignments, it was necessary to perform multiple runs for each dataset. An initial run of 5×10^6^ generations was performed and parameter estimates (nucleotide frequencies, transition/transversion ratio [κ], gamma shape parameter [α]) taken from this run. Further runs of 5×10^6^ generations were started using the fixed values of parameters from the first run and the highest likelihood tree from the previous run until convergence of the three separate chains was visibly seen. Posterior consensus trees were generated using the final run only (after a burn-in of 50%), summarising 25000 trees.

In order to isolate probable epidemiologically linked UK-restricted transmission clusters from phylogenetically-defined clusters of ≥10 individuals, clusters were included in a new phylogenetic analysis together with the 100 most closely related sequences to the cluster. These were selected from the LANL HIV database by comparing the cluster consensus sequence to all sequences for that subtype in the database by % difference. For each cluster and its most closely related global sequences, a MrBayes tree was generated (5×10^6^ generations, HKY+Γ model). Clusters that remained monophyletic with high support (≥0.95) were then defined as UK transmission clusters.

### Time-scaled phylogenies

Dated phylogenies were obtained using a Bayesian MCMC method (BEAST version 1.4.7; [13] using a relaxed molecular clock [25]. Clades of ≥10 individuals fully supported in MrBayes phylogenetic trees were analysed in their entirety and the results for subtrees defined as UK transmission clusters extracted from the full time-scaled trees. The date used for each sequence in any analysis was the number of days since the isolation date of the oldest sequence within the entire dataset, with sequences dated using the number of days from the earliest sequence isolation date. Analysis was performed using the SRD06 model of nucleotide substitution [26] with a lognormal distribution of rates amongst branches. Lognormal priors were placed on root height, corresponding to a median of 20 years and an upper 5% limit of 40 years. The most appropriate demographic prior on population size (constant or exponentially increasing) was determined using Bayes factors [27] with a log_10_ Bayes factor ≥5 indicative of substantial evidence of improved model fitting [28]. For each cluster, BEAST analysis used three separate MCMC runs of chain length 1×10^7^ (sampling parameters and trees every 1000 generations) combined after a 10% burn-in (leaving 30000 trees). Tree samples were used to generate a maximum clade credibility (MCC) tree with mean node heights using TreeAnnotator (available from http://beast.bio.ed.ac.uk).

### Sequences

Partial sequences of the HIV pol gene analyzed here have been deposited in GenBank under accession numbers GQ462027-GQ462532.

## Supporting Information

Table S1 CD4 count at treatment by HIV subtype. ^1^ Data from UK CHIC [23]. ^2^ Mean of the corrected CD4 count distribution (see Text S1). ^3^ Mann-Whitney U test.
(0.07 MB PDF)

Figure S1 Bayesian MCMC phylogeny of closely related subtype A sequences. Fully-supported clades of ≥10 patients are shown in red. Scale bar shows the number of substitutions (N = 367).
(0.04 MB PDF)

Figure S2 Bayesian MCMC phylogeny of closely related subtype C sequences. Fully-supported clades of ≥10 patients are shown in red. Scale bar shows the number of substitutions (N = 1372).
(0.10 MB PDF)

Figure S3 Bayesian MCMC phylogeny of closely related sequences of other non-B subtypes. Fully-supported clades of ≥10 patients are shown in red. Scale bar shows the number of substitutions (N = 1035).
(0.07 MB PDF)

Figure S4 Time-scaled phylogenies of clusters of ≥10 patients from other non-B subtypes. Red dots indicate UK transmission clusters as defined against analysis with global diversity. Grey lines indicate non UK-based segments of the phylogeny, black lines indicate UK-based lineages. The scale bar indicates calendar years.
(0.04 MB PDF)

Figure S5 Sensitivity analysis of goodness of fit to power law distribution with respect to time depth of clusters. Variation in shape parameter, γ (negative gradient in log-log space) and R^2^ (pearson correlation) of power law best fits as a function of time depth of the networks. Networks are formed from individuals assumed to be in contact within UK-based clusters, if the time to the Most Recent Common Ancestor (TMRCA) of their virus sequences does not exceed a specified time depth. The graph shows the best fit to a power law comes from networks with a time depth of 5–6 years (R^2^ = 0.95, γ = 2.1). Time depths of <3 years do not capture enough links between individuals, and time depths of >8 years result in too many connections for a good fit to a power law.
(0.04 MB PDF)

Figure S6 Time-scaled phylogenies of subtype A clusters of size ≥10 with terminal branches removed. Red nodes indicate UK transmission clusters as defined against analysis with global diversity. Black/grey nodes indicate where terminal branches have been removed. The scale bar is in calendar years.
(0.04 MB PDF)

Figure S7 Time-scaled phylogenies of subtype C clusters of size ≥10 with terminal branches removed. Red nodes indicate UK transmission clusters as defined against analysis with global diversity. Black/grey nodes indicate where terminal branches have been removed. The scale bar is in calendar years.
(0.05 MB PDF)

Figure S8 Time-scaled phylogenies of other non-B subtype clusters of size ≥10 with terminal branches removed. Red nodes indicate UK transmission clusters as defined against analysis with global diversity. Black/grey nodes indicate where terminal branches have been removed. The scale bar is in calendar years.
(0.05 MB PDF)

Figure S9 Histograms of CD4 counts by HIV subtype. (A–D) Distribution of first available CD4 count (“Diagnosis”) by subtype. (E–H) Distribution of CD4 count at first treatment (Treatment”) by subtype. (I–L) Distribution of CD4 count at treatment after correction by subtype. (M–N) Combined distributions at diagnosis (M) and first treatment (N).
(0.11 MB PDF)

Figure S10 Recruitment to the UK HIV Drug Resistance Database. Number of individuals recruited to the UK HI Drug Resistance Database by year, according to treatment status at recruitment. Naïve: recruited with HIV genotype assay taken before initiation of therapy (to identify transmitted drug resistance). Experienced: recruited with genotype assay performed due to failure of existing antiretroviral therapy.
(0.05 MB PDF)

Text S1 (0.10 MB PDF)
