# Supplementary material for: Molecular Phylodynamics of the Heterosexual HIV Epidemic in the United Kingdom
Source: PLoS Pathog. 2009 Sep 25;5(9):e1000590. doi: 10.1371/journal.ppat.1000590 (PMC2742734; doi:10.1371/journal.ppat.1000590)
Supplement: Table S1 — CD4 count at treatment by HIV subtype. 1 Data from UK CHIC [23]. 2 Mean of the corrected CD4 count distribution (see Text S1). 3 Mann-Whitney U test. (0.07 MB PDF) [file ppat.1000590.s001.pdf]

Table S1. CD4 count at treatment by HIV subtype[1]

	N	Mean (95% CI)[2]	Comparison with B[3]
A	397	219 (207 – 231)	$Z = -1.621$
			$P = 0.105$
C	816	205 (197 – 213)	$Z = -4.111$
			**$P < 0.001$**
others	659	240 (230 – 250)	$Z = -1.169$
			$P = 0.242$
B	892	228 (220 – 236)	-

[1] Data from UK CHIC[23]

[2] Mean of the corrected CD4 count distribution (see Text S1)

[3] Mann-Whitney U test
