# Supplementary figures and images for: Molecular Phylodynamics of the Heterosexual HIV Epidemic in the United Kingdom
Source: PLoS Pathog. 2009 Sep 25;5(9):e1000590. doi: 10.1371/journal.ppat.1000590 (PMC2742734; doi:10.1371/journal.ppat.1000590)

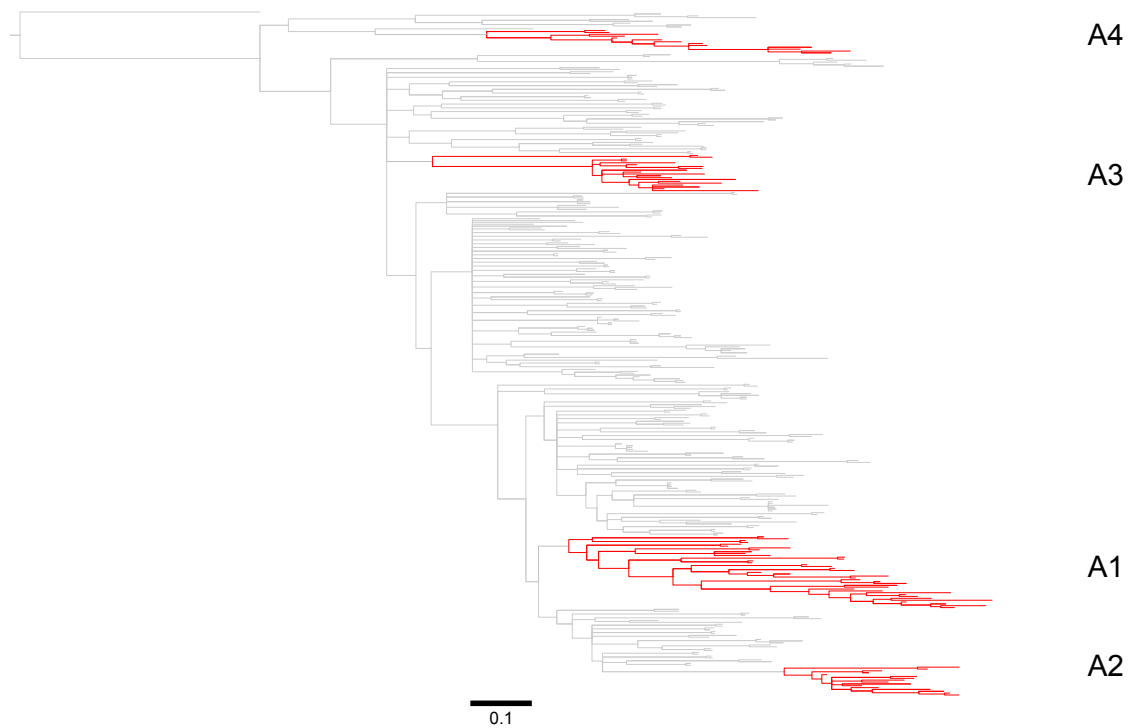

**Figure S1**

Supplement: Figure S1 — Bayesian MCMC phylogeny of closely related subtype A sequences. Fully-supported clades of ≥10 patients are shown in red. Scale bar shows the number of substitutions (N = 367). (0.04 MB PDF) [file ppat.1000590.s002.pdf]

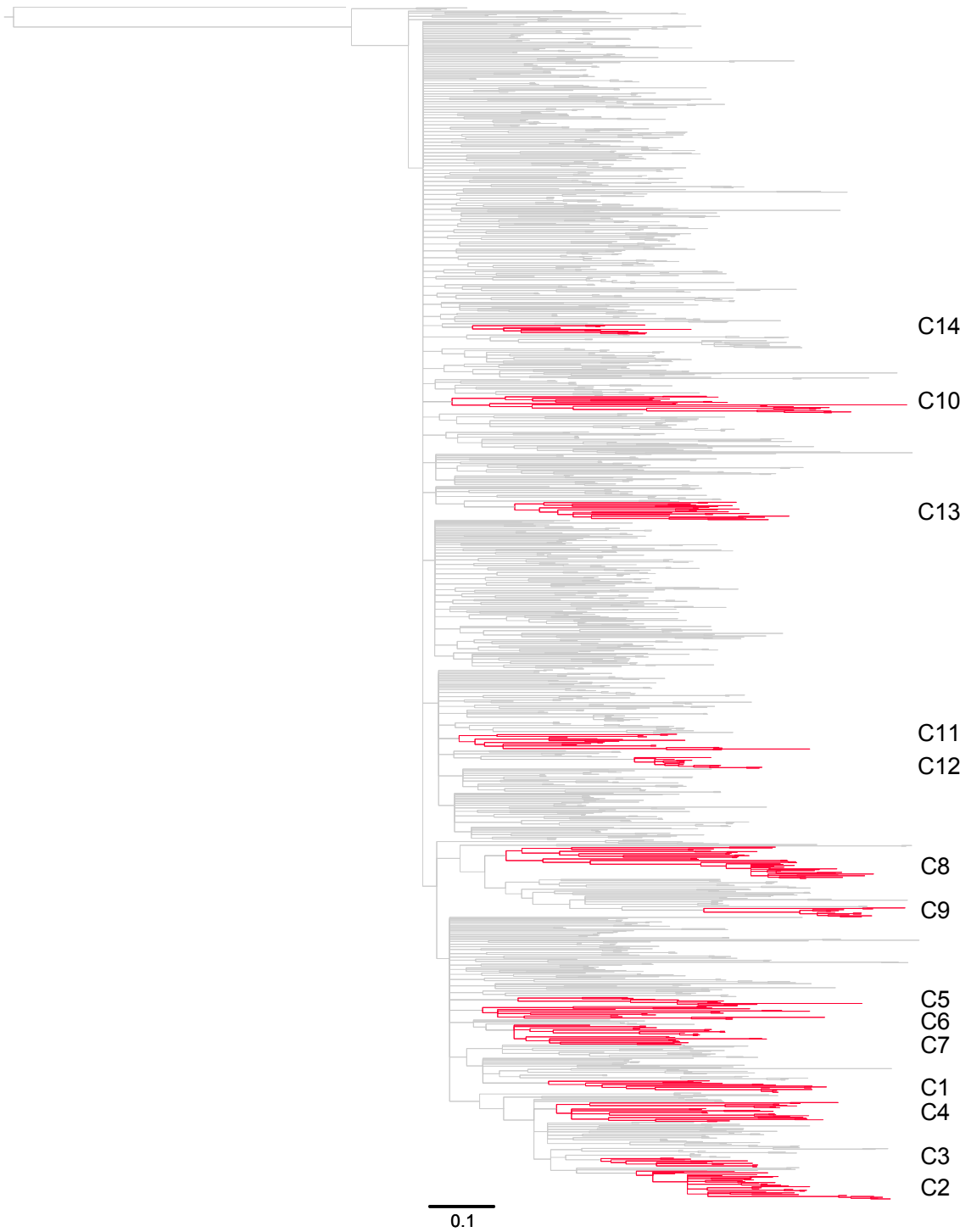

**Figure S2**

Supplement: Figure S2 — Bayesian MCMC phylogeny of closely related subtype C sequences. Fully-supported clades of ≥10 patients are shown in red. Scale bar shows the number of substitutions (N = 1372). (0.10 MB PDF) [file ppat.1000590.s003.pdf]

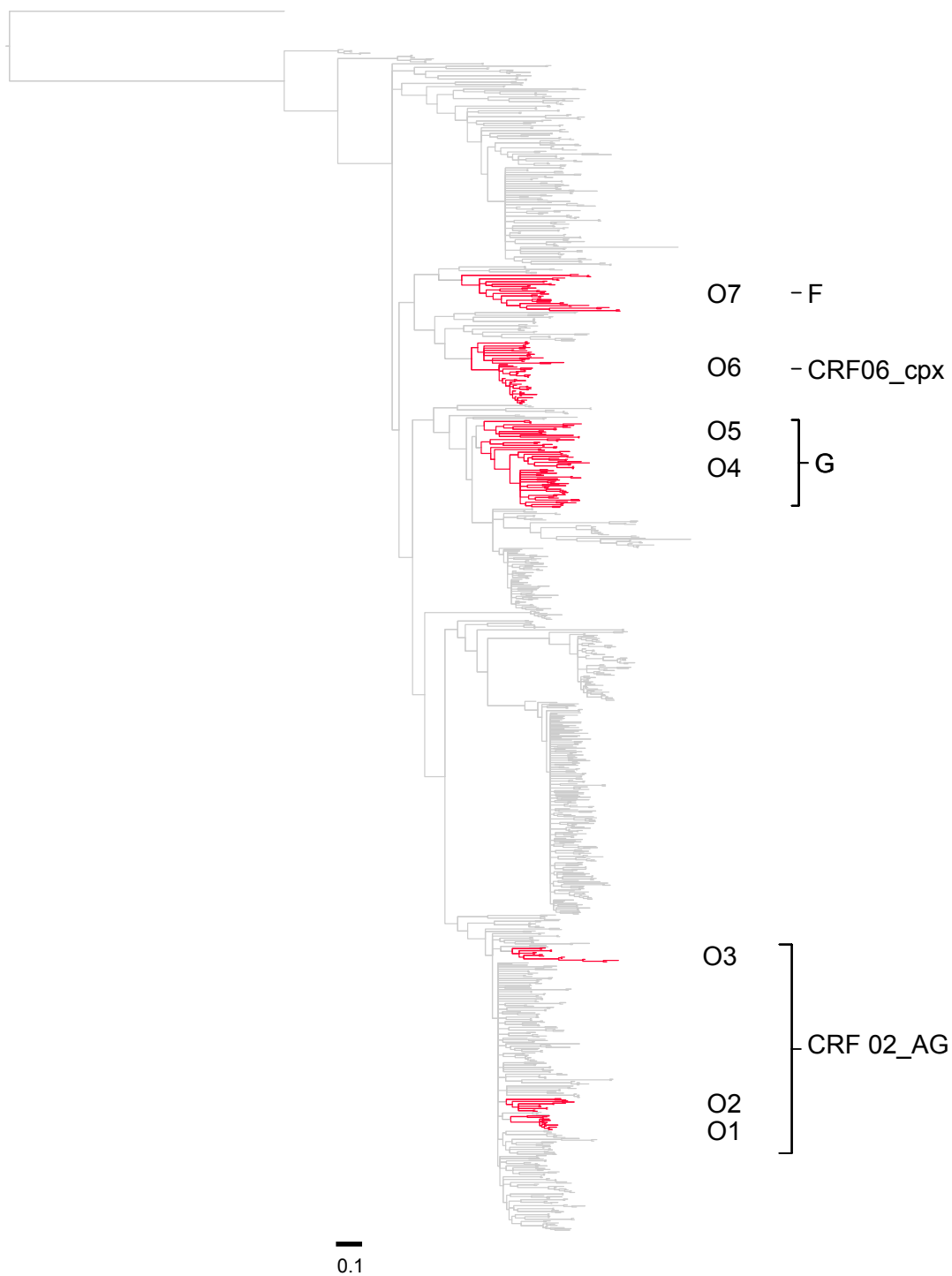

**Figure S3**

Supplement: Figure S3 — Bayesian MCMC phylogeny of closely related sequences of other non-B subtypes. Fully-supported clades of ≥10 patients are shown in red. Scale bar shows the number of substitutions (N = 1035). (0.07 MB PDF) [file ppat.1000590.s004.pdf]

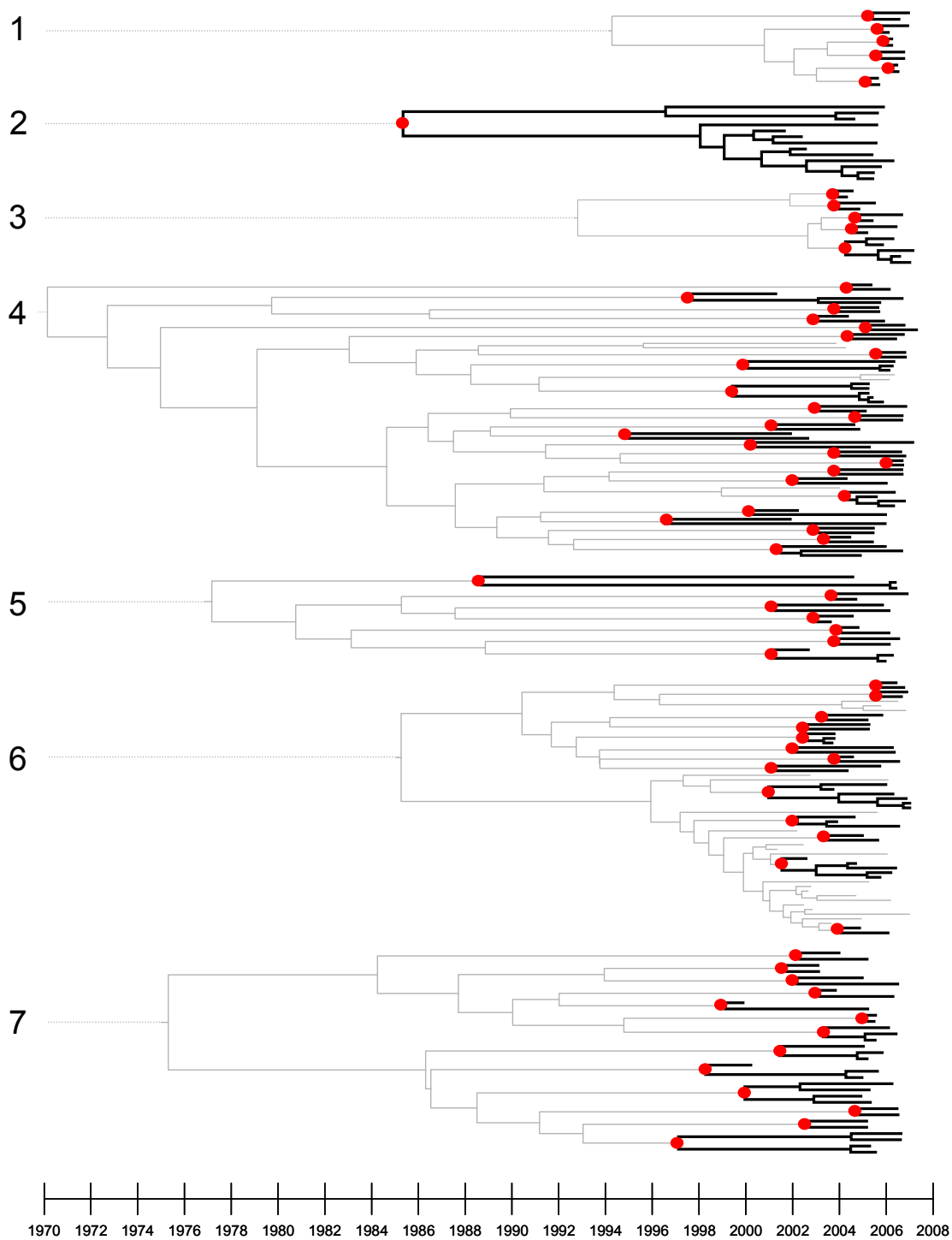

**Figure S4**

Supplement: Figure S4 — Time-scaled phylogenies of clusters of ≥10 patients from other non-B subtypes. Red dots indicate UK transmission clusters as defined against analysis with global diversity. Grey lines indicate non UK-based segments of the phylogeny, black lines indicate UK-based lineages. The scale bar indicates calendar years. (0.04 MB PDF) [file ppat.1000590.s005.pdf]

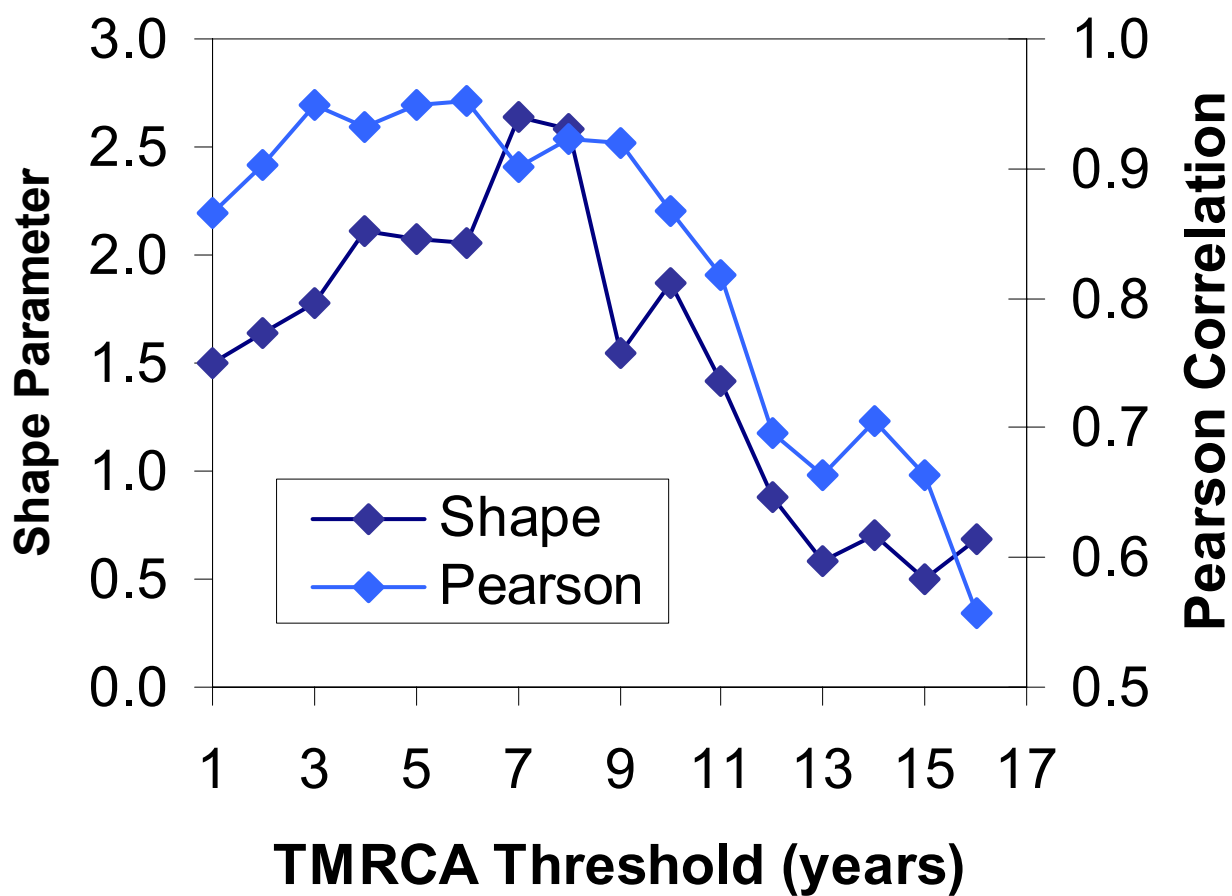

**Figure S5**

Supplement: Figure S5 — Sensitivity analysis of goodness of fit to power law distribution with respect to time depth of clusters. Variation in shape parameter, γ (negative gradient in log-log space) and R2 (pearson correlation) of power law best fits as a function of time depth of the networks. Networks are formed from individuals assumed to be in contact within UK-based clusters, if the time to the Most Recent Common Ancestor (TMRCA) of their virus sequences does not exceed a specified time depth. The graph shows the best fit to a power law comes from networks with a time depth of 5–6 years (R2 = 0.95, γ = 2.1). Time depths of <3 years do not capture enough links between individuals, and time depths of >8 years result in too many connections for a good fit to a power law. (0.04 MB PDF) [file ppat.1000590.s006.pdf]

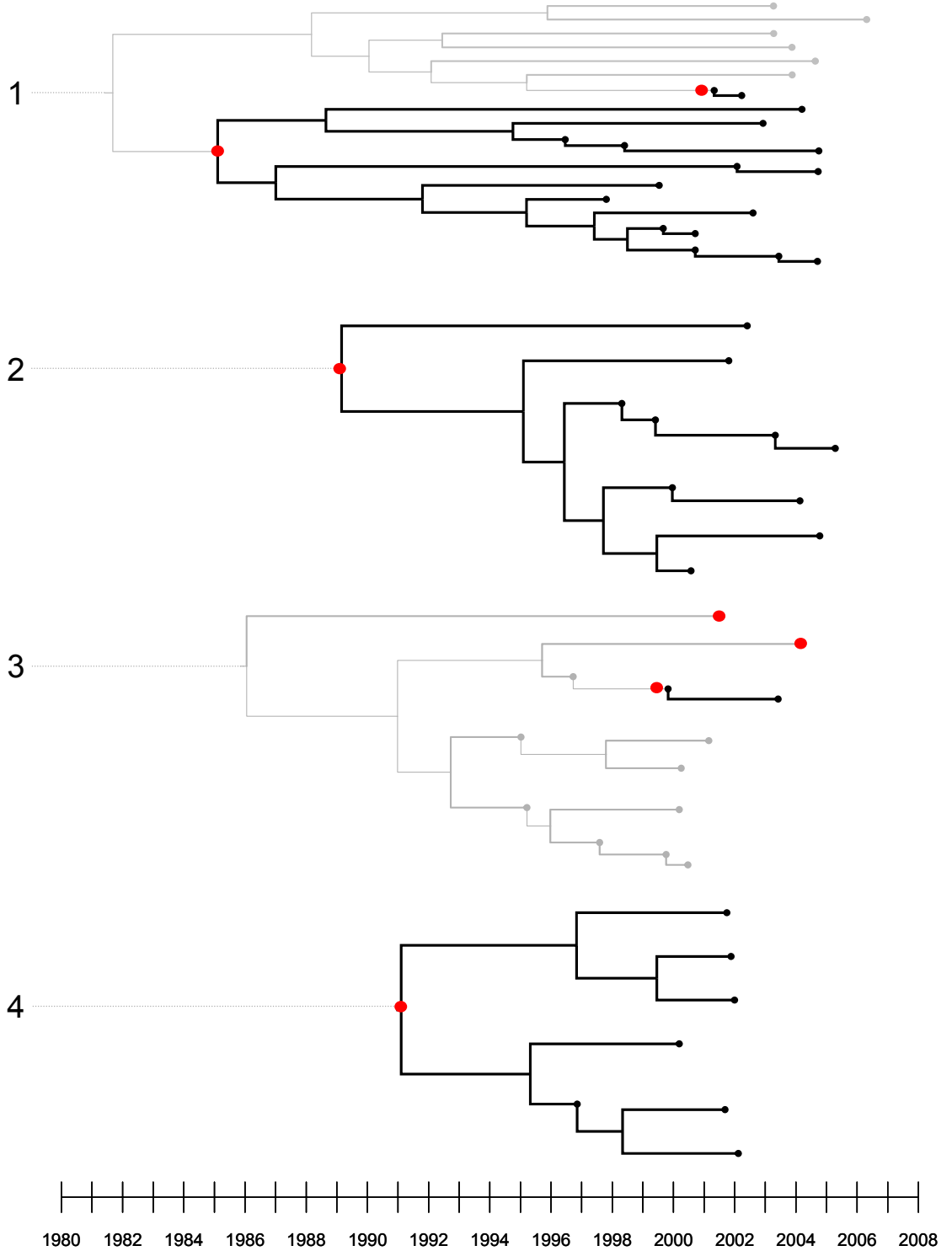

**Fig S6**

Supplement: Figure S6 — Time-scaled phylogenies of subtype A clusters of size ≥10 with terminal branches removed. Red nodes indicate UK transmission clusters as defined against analysis with global diversity. Black/grey nodes indicate where terminal branches have been removed. The scale bar is in calendar years. (0.04 MB PDF) [file ppat.1000590.s007.pdf]

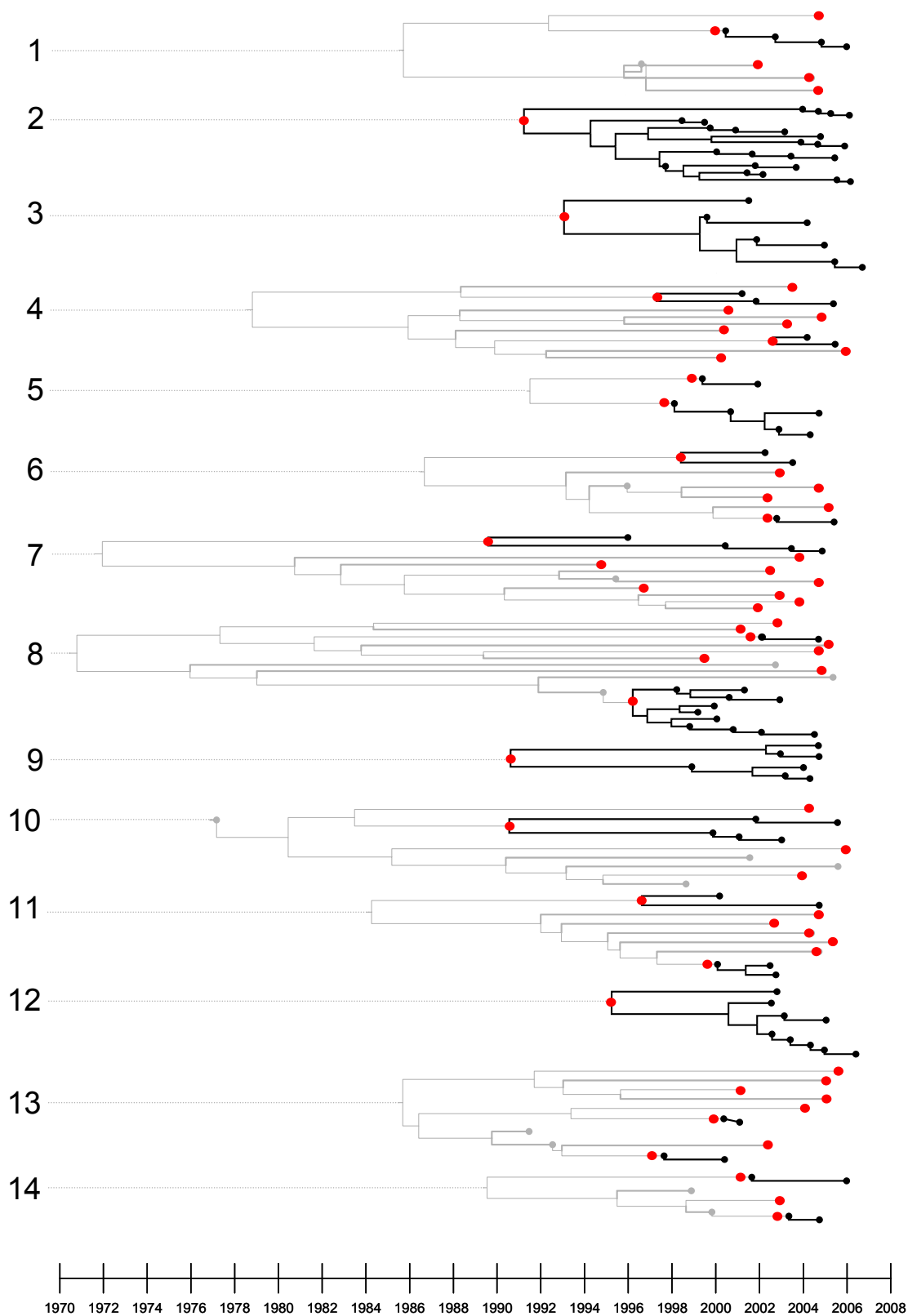

**Figure S7**

Supplement: Figure S7 — Time-scaled phylogenies of subtype C clusters of size ≥10 with terminal branches removed. Red nodes indicate UK transmission clusters as defined against analysis with global diversity. Black/grey nodes indicate where terminal branches have been removed. The scale bar is in calendar years. (0.05 MB PDF) [file ppat.1000590.s008.pdf]

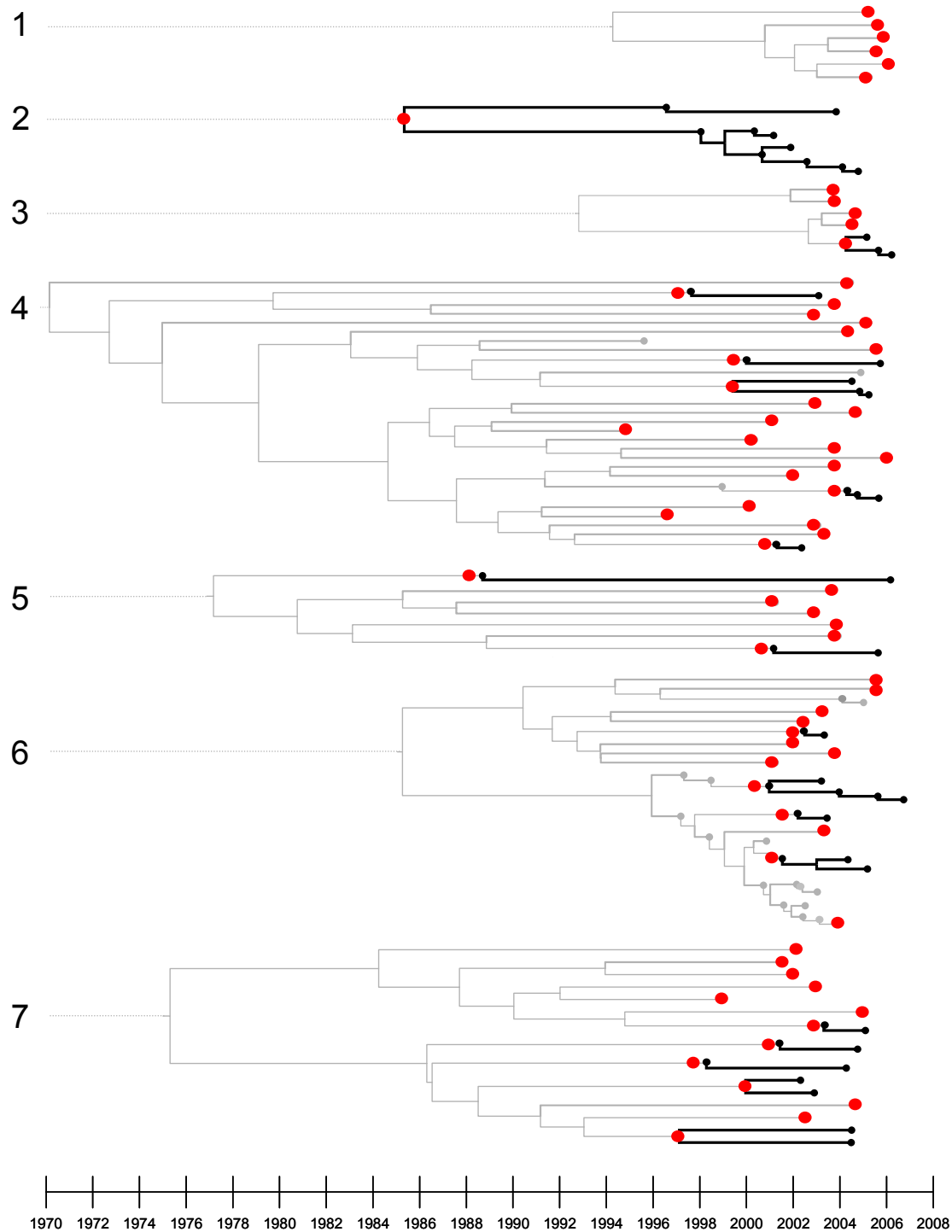

**Figure S8**

Supplement: Figure S8 — Time-scaled phylogenies of other non-B subtype clusters of size ≥10 with terminal branches removed. Red nodes indicate UK transmission clusters as defined against analysis with global diversity. Black/grey nodes indicate where terminal branches have been removed. The scale bar is in calendar years. (0.05 MB PDF) [file ppat.1000590.s009.pdf]

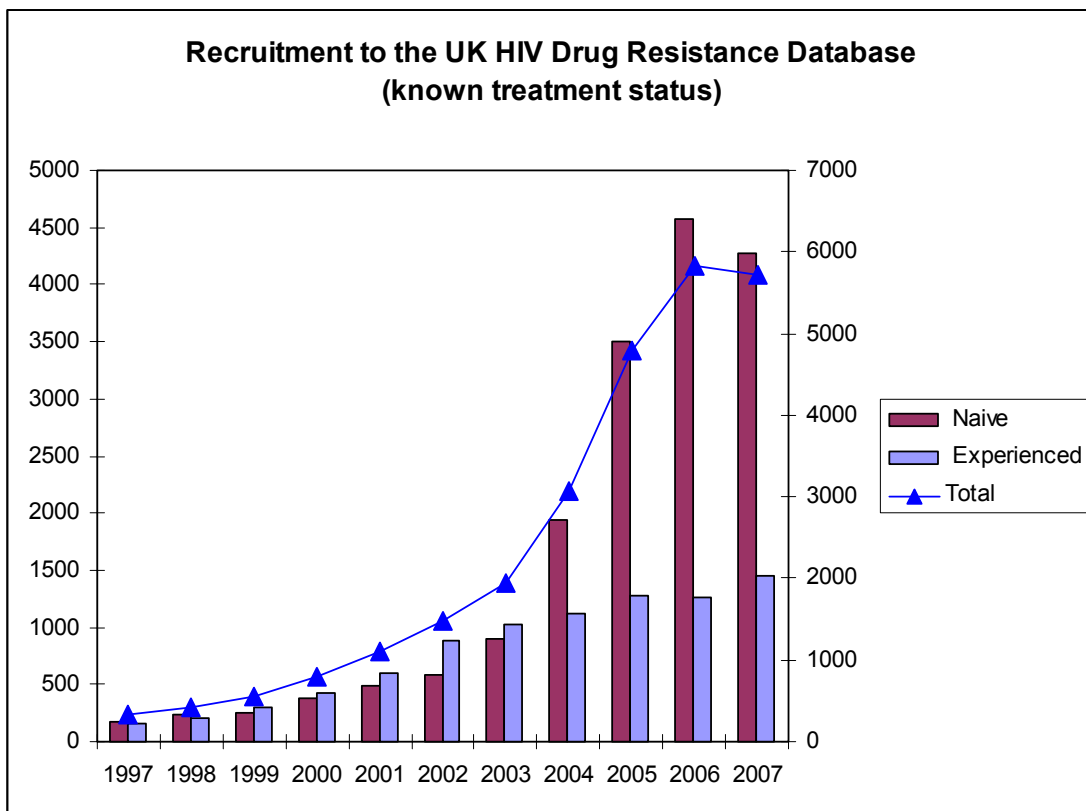

**Figure S10**

Supplement: Figure S10 — Recruitment to the UK HIV Drug Resistance Database. Number of individuals recruited to the UK HI Drug Resistance Database by year, according to treatment status at recruitment. Naïve: recruited with HIV genotype assay taken before initiation of therapy (to identify transmitted drug resistance). Experienced: recruited with genotype assay performed due to failure of existing antiretroviral therapy. (0.05 MB PDF) [file ppat.1000590.s011.pdf]
