# Supplementary material for: Molecular Phylodynamics of the Heterosexual HIV Epidemic in the United Kingdom
Source: PLoS Pathog. 2009 Sep 25;5(9):e1000590. doi: 10.1371/journal.ppat.1000590 (PMC2742734; doi:10.1371/journal.ppat.1000590)
Supplement: Figure S9 — Histograms of CD4 counts by HIV subtype. (A–D) Distribution of first available CD4 count (“Diagnosis”) by subtype. (E–H) Distribution of CD4 count at first treatment (Treatment”) by subtype. (I–L) Distribution of CD4 count at treatment after correction by subtype. (M–N) Combined distributions at diagnosis (M) and first treatment (N). (0.11 MB PDF) [file ppat.1000590.s010.pdf]

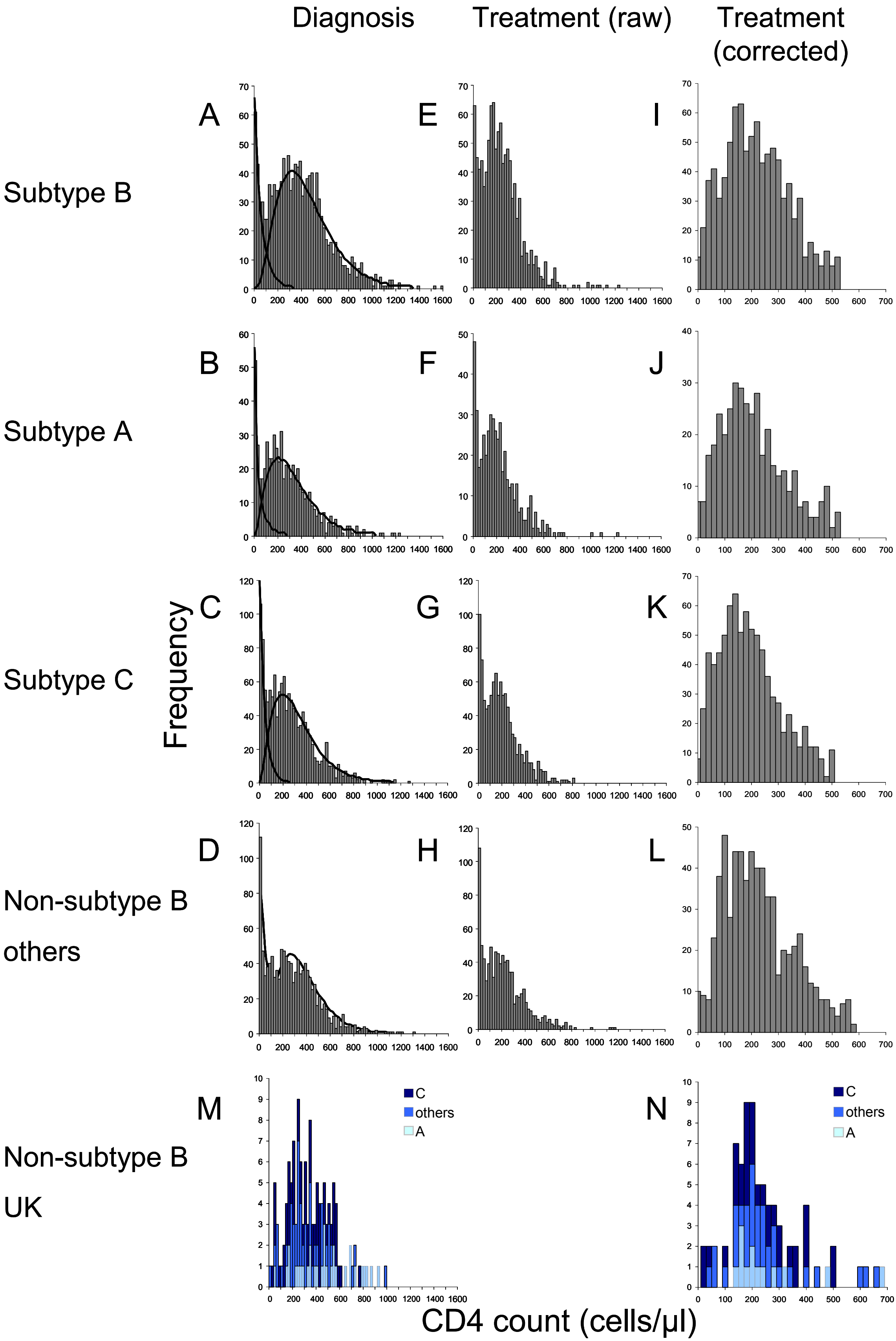
Diagnosis
Treatment (raw)
Treatment (corrected)
Subtype B
Subtype A
Subtype C
Non-subtype B others
Non-subtype B UK
Frequency
A
B
C
D
E
F
G
H
I
J
K
L
M
N
C
others
A
CD4 count (cells/μl)

Figure S9
