# Supplementary material for: Molecular Phylodynamics of the Heterosexual HIV Epidemic in the United Kingdom
Source: PLoS Pathog. 2009 Sep 25;5(9):e1000590. doi: 10.1371/journal.ppat.1000590 (PMC2742734; doi:10.1371/journal.ppat.1000590)
Supplement: Text S1 — (0.10 MB PDF) [file ppat.1000590.s012.pdf]

SUPPORTING TEXT

Methods

Recruitment to the UK HIV Drug Resistance Database

Individuals recruited to this database over a period of 10 years have been included in this study. The distribution of numbers recruited by year (1997-2007) is shown in Figure S10 for antiretroviral treatment-naïve and -treated individuals, respectively.

CD4 count analysis

In order to compare CD4 counts between groups, a mixture model of two gamma distributions was fitted to the first available CD4 count for each patient using the function *mix* from the library *mixdist* implemented using R (www.R-project.org). Two overlapping component gamma distributions were fitted with initial mean values of 40 and 300 and initial standard deviations estimated from the full dataset for each subtype. The fit of each mixture model was compared to that of a single gamma distribution using the reported chi-squared statistic.

Results – CD4 count distributions at diagnosis and first treatment

The median first CD4 counts by subtype were as follows: B, 356 (n=1288); A, 230 (n=600); C, 219 (n=1326); others, 255 (n=1152). The number of patients diagnosed very late (first CD4 count ≤50) are higher for non-B subtypes (A: 15%, C: 16%, others: 15%, B: 9%) and these numbers are reflected in the numbers of CD4 counts ≤50 at therapy (A: 18%, C: 21%, others: 20%, B: 11%) (Fig. S9 E-H).

Two gamma distributions were fitted to the whole CD4 count distributions at diagnosis as this provided a substantially better fit than a single gamma distribution for all subtypes (two distributions, A: P = 0.9973, B: P = 0.8892, C: P = 0.9508,

others: P = 0.954; one distribution, A: P = 0.1168, B: P = <0.0001, C: P = 0.0002, others: P = <0.0001) (Fig S9 A-D). Taking the 1st percentile of the first gamma distribution as a threshold to exclude very late diagnoses and the 95th percentile of the trimmed distribution to exclude high CD4 count outliers, reveals very similar distributions of CD4 counts at treatment (Fig S9 I-L). When we consider that part of the non-B dataset that comprises UK-based transmissions, the proportion having very low CD4 counts at diagnosis (4%) and at treatment (5%) (Fig S9 M-N) is considerably reduced compared to the entire dataset. The distribution of CD4 counts at treatment for these patients is very similar to the corrected CD4 count distributions for the full datasets (Figure S9 I-L, N), suggesting it is the immigrant fraction that is responsible for the lower CD4 count at diagnosis, and that risk factor for infection does not influence CD4 count at diagnosis for individuals infected in the UK. Overall, after correcting for outliers, the distributions of CD4 counts at treatment are similar for all three subtypes. Only subtype C shows a statistically significantly different mean and distribution to that of subtype B-infected patients (Table S1) of about 10% with a mean of 205 cells/μl as compared to 228 cells/μl (subtype B).

The UK HIV Drug Resistance Database

Steering Committee:

Jane Anderson, Homerton University Hospital, London; David Asboe and Anton Pozniak, Chelsea & Westminster Hospital, London; Sheila Burns, Royal Infirmary of Edinburgh; Sheila Cameron, Gartnavel General Hospital, Glasgow; Patricia Cane, Health Protection Agency, Porton Down; Ian Chrystie, Guy's and St. Thomas' NHS Foundation Trust, London; Duncan Churchill, Brighton and Sussex University Hospitals NHS Trust; Duncan Clark, St Bartholomew's and The London NHS Trust; Valerie Delpech and Deenan Pillay, Health Protection Agency, Centre for Infections, London; Linda Lazarus, Expert Advisory Group on AIDS Secretariat, Health Protection Agency, London; David Dunn, Esther Fearnhill, Hannah Green and Kholoud Porter, MRC Clinical Trials Unit, London; Philippa Easterbrook and Mark Zuckerman, King's College Hospital, London; Anna Maria Geretti, Royal Free NHS Trust, London; Paul Kellam, Deenan Pillay, Andrew Phillips and Caroline Sabin, Royal Free and University College Medical School, London; David Goldberg, Health Protection Scotland, Glasgow; Mark Gompels, Southmead Hospital, Bristol; Antony Hale, Leeds Teaching Hospitals NHS Trust; Steve Kaye, St. Mary's Hospital, London; Svilen Konov, Community Advisory Board; Andrew Leigh-Brown, University of Edinburgh; Nicola Mackie, St. Mary's Hospital, London; Chloe Orkin, St. Bartholomew's Hospital, London; Erasmus Smit, Health Protection Agency, Birmingham Heartlands Hospital; Peter Tilston, Manchester Royal Infirmary; Ian Williams, Mortimer Market Centre, London; Hongyi Zhang, Addenbrooke's Hospital, Cambridge

Participating laboratories:

Addenbrooke's Hospital, Cambridge (Hongyi Zhang); Department of Virology, St Bartholomew's and The London NHS Trust (Duncan Clark, Ines Ushiro-Lumb, Tony Oliver, David Bibby); Belfast Health and Social Care Trust (Suzanne Mitchell); HPA Birmingham Public Health Laboratory (Erasmus Smit); Chelsea and Westminster Hospital, London (Adrian Wildfire); Dulwich Hospital, London (Melvyn Smith); Royal Infirmary of Edinburgh (Jill Shepherd); West of Scotland Specialist Virology Lab Gartnavel, Glasgow (Alasdair MacLean); Guy's and St. Thomas' NHS Foundation Trust, London (Ian Chrystie); Leeds Teaching Hospitals NHS Trust (Diane Bennett); Specialist Virology Centre, Liverpool (Mark Hopkins) and Manchester (Peter Tilston); Department of Virology at Royal Free Hospital, London (Clare Booth, Ana Garcia-Diaz); St Mary's Hospital, London (Steve Kaye); University College London Hospitals (Stuart Kirk)

Coordinating Centre:

Medical Research Council Clinical Trials Unit (MRC CTU), London (David Dunn, Esther Fearnhill, Hannah Green, Kholoud Porter, Kate Coughlin).
